# Heterogeneity characterization of hepatocellular carcinoma based on the sensitivity to 5-fluorouracil and development of a prognostic regression model

**DOI:** 10.3389/fphar.2023.1252805

**Published:** 2023-09-07

**Authors:** Xinyu Gu, Shuang Li, Xiao Ma, Di Huang, Penghui Li

**Affiliations:** ^1^ Department of Oncology, The First Affiliated Hospital of Henan University of Science and Technology, Luoyang, China; ^2^ Hematology Department, Traditional Chinese Hospital of Luan, Lu’an, China; ^3^ School of Medicine, Zhejiang University, Hangzhou, China; ^4^ Department of Child Health Care, The Third Affiliated Hospital of Zhengzhou University, Zhengzhou, China; ^5^ The Department of General Surgery, The First Affiliated Hospital of Henan University of Science and Technology, Luoyang, China

**Keywords:** 5-fluorouracil, hepatocellular carcinoma, machine learning, genomic variation, immune infiltration, prognosis

## Abstract

**Background:** 5-Fluorouracil (5-FU) is a widely used chemotherapeutic drug in clinical cancer treatment, including hepatocellular carcinoma (HCC). A correct understanding of the mechanisms leading to a low or lack of sensitivity of HCC to 5-FU-based treatment is a key element in the current personalized medical treatment.

**Methods:** Weighted gene co-expression network analysis (WGCNA) was used to analyze the expression profiles of the cancer cell line from GDSC2 to identify 5-FU-related modules and hub genes. According to hub genes, HCC was classified and the machine learning model was developed by ConsensusClusterPlus and five different machine learning algorithms. Furthermore, we performed quantitative reverse transcription-polymerase chain reaction (qRT-PCR) analysis on the genes in our model.

**Results:** A total of 19 modules of the cancer cell line were divided by WGCNA, and the most negative correlation with 5-FU was the midnight blue module, from which 45 hub genes were identified. HCC was divided into three subgroups (C1, C2, and C3) with significant overall survival (OS) differences. OS of C1 was the shortest, which was characterized by a high clinical grade and later T stage and stage. OS of C3 was the longest. OS of C2 was between the two subtypes, and its immune infiltration was the lowest. Five out of 45 hub genes, namely, *TOMM40L*, *SNRPA*, *ILF3*, *CPSF6*, and *NUP205*, were filtered to develop a risk regression model as an independent prognostic indicator for HCC. The qRT-PCR results showed that *TOMM40L*, *SNRPA*, *ILF3*, *CPSF6*, and *NUP205* were remarkably highly expressed in hepatocellular carcinoma.

**Conclusion:** The HCC classification based on the sensitivity to 5-FU was in line with the prognostic differences observed in HCC and most of the genomic variation, immune infiltration, and heterogeneity of pathological pathways. The regression model related to 5-FU sensitivity may be of significance in individualized prognostic monitoring of HCC.

## Introduction

Hepatocellular carcinoma (HCC) is a critical global healthcare issue with a mortality-to-morbidity ratio as high as 91.6% (Villarruel-Melquiades et al., 2023). Patients with HBV/HCV infection, cirrhosis of any cause, alcoholism, non-alcoholic steatohepatitis, or family history of HCC are considered high-risk groups for HCC, especially among men aged over 40 years old (Xie et al., 2023). Surgical treatment, including hepatectomy and orthotopic liver transplantation, is widely used for tumor eradication (Kawaguchi et al., 2016), but it is also restricted by the applicability of patients and the availability of organs (Koza et al., 2023). In a clinical practice, more than 60% of HCC cases are diagnosed in the late stage, suggesting possible missed diagnostic opportunity. At present, the best choice for advanced HCC is systemic treatment, with sorafenib and lenvatinib as the first choice (Yang et al., 2023). However, patients treated with drugs will have to face the problem of drug resistance after drug treatment. Improving the understanding of the mechanism of HCC resistance is expected to bring further benefits to patients.

As a synthetic fluorinated pyrimidine analog, 5-fluorouracil (5-FU) enters cells as an anti-metabolite, imitates molecules vital to cell growth, interferes with basic biosynthetic activity by inhibiting the effect of thymidylate synthase (TS), or mistakenly mixes its metabolites into DNA and RNA, thereby inducing cytotoxicity (Blondy et al., 2020; Mafi et al., 2023). Since its approval by FDA in 1962, 5-FU has been widely applied alone or together with other drugs in treating various cancers, such as advanced head and neck squamous cell carcinoma (Yamauchi et al., 2023), colorectal cancer (Wosiak et al., 2023), gastric cancer (Kang et al., 2014), and metastatic breast cancer (Karapetis et al., 1999; Holmes et al., 2018). 5-Fu is also a widely used chemotherapeutic drug for patients with HCC. There is an urgent need to better improve the sensitivity of HCC to chemotherapy (Hu et al., 2016), and an accurate understanding of mechanisms that contribute to a lack of or a low sensitivity of HCC to 5-FU-based treatment is a critical component of the current trend of individualized medical care. Identifying and confirming current 5-FU-based predictive biomarkers, as well as developing novel targeted medicines for HCC therapy, may enhance patients’ prognoses in the future (Vodenkova et al., 2020).

In this study, genes related to 5-Fu sensitivity were screened from large data sets for identification of HCC, in order to characterize the heterogeneity of HCC from molecular aspects and tumor microenvironment (TME). Genes suitable for constructing a risk model were identified from those related to 5-Fu sensitivity, hoping to provide a promising target for understanding 5-FU resistance of HCC.

## Materials and methods

### Data source

Clinical data and RNA sequencing of HCC were downloaded from the LIHC project of The Cancer Genome Atlas (TCGA) database (https://cancergenome.nih.gov), and a total of 50 corresponding paracancerous tissues and 365 HCC tumor tissues were incorporated into the analysis. A set of HCC chip data numbered GSE14520 were collected from the Gene Expression Omnibus (GEO) database (https://www.ncbi.nlm.nih.gov/geo/). Another set of data were downloaded from the HCCDB database (http://lifeome.net/database/hccdb/download.html), which provides expression profiles of HCC samples. Meanwhile, pan-cancer cell line drug sensitivity and genomic data resources were acquired from the Genomics of Drug Sensitivity in Cancer (GDSC) database (https://www.cancerrxgene.org/) (Yang et al., 2013).

### Weighted gene co-expression network analysis (WGCNA)

WGCNA was performed for analyzing the expression profiles of cancer cell lines downloaded from GDSC2. Samples were clustered and used to construct a gene co-expression network, from which modules were identified and then related to external data. Key drivers in the “WGCNA” package (Langfelder and Horvath, 2008) of R were analyzed based on the relationships among the module. Under the selected parameters, the sampleTree function provided by “WGCNA” was used to cluster the HCC cell lines and present the outliers. The gene expression matrices of the retained samples were extracted, and Pearson correlation was computed to calculate the correlation between twisted genes. Under different power values, we selected the optimal soft threshold β by analyzing the scale independence and average connectivity of modules using the “pickSoftThreshold” function provided by the “WGCNA” package. The “scaleFreePlot” function was adopted to evaluate whether the topology of the network was scale-free. The hierarchical clustering of genes was implemented by using the “hclust” function. The distance clustering threshold (height = 0.25, deepSplit = 3) was set by the cutreeDynamic function in the dynamicTreeCut package, and the minimum number of genes was 30 in each module. The automatic module merge step was performed using the mergeCloseModules function. The interested modules were the most relevant to 5-fluorouracil; therefore, the IC_50_ value of 5-fluorouracil for module–trait relationships was analyzed.

### Unsupervised clustering on HCC

The “limma” package (Ritchie et al., 2015) was employed to discriminate differentially expressed genes (DEGs) meeting FDR<0.05 and log2(Fold Change) > 1. DEGs were then screened by overlapping analysis with gene modules associated with 5-fluorouracil. The “ConsensusClusterPlus” package was applied to run the consensus clustering on the TCGA-LIHC data matrix (Wilkerson and Hayes, 2010). The initial step was to subsample 80% items and features. Each subsample was then partitioned into k groups. Afterward, consensus values were calculated and stored in a consensus matrix for each k-value. The output graphical plots included the consensus matrix plot and the empirical cumulative distribution function (CDF) plot.

## Single-nucleotide variant (SNV) and copy-number variant (CNV) analyses

### Genomic variation analysis

Genomic variation includes small insertions or deletions (indels), single-nucleotide variants, and CNVs. SNVs and CNVs, all belong to the category of genomic variation. After reading the MAF file from TCGA-LIHC, the generated MAF object was passed to the “maftools” package (Mayakonda et al., 2018) for SNV analysis and oncoplot drawing. GISTIC 2.0, which calculates a statistic involving the occurrence frequency and distortion amplitude, was employed to analyze CNV data. The characteristic of this method is to identify the regions of the genome, where anomalies occur more frequently than accidentally expected and gives more weight to high-amplitude events (high-level copy number gain or homozygous deletion) that are unlikely to represent random distortions. For each important region, the method defines a “peak region” with a maximum aberration frequency and amplitude (Beroukhim et al., 2007).

### Immune cell infiltration analysis

The ESTIMATE algorithm, which leverages the properties of the TCGA-LIHC transcriptional profiles to infer the degrees of stromal and immune cell infiltration, was applied to determine the ESTIMATE score (Yoshihara et al., 2013). Different methods for assessing the level of immune infiltration, including CIBERSORT, ssGSEA, MCPcounter, and TIMER, were properly applied. CIBERSORT was used to measure the intra-sample (within-leukocyte) proportions of immune cell populations (Newman et al., 2015). Different from CIBERSORT, MCPcounter outputs the estimated abundance of each cell population, thereby enabling a comparison between samples to be expressed in arbitrary units (Becht et al., 2016). TIMER takes tissue specificity into account when estimating immune cell populations (Li et al., 2017), and this method helps identify associations between six types of immune cell infiltration and clustering in the TCGA-LIHC cohort.

### Establishment of a risk stratification tool using multiple machine learning analysis

Univariate COX regression analysis identified prognostic genes from the intersection of DEGs and 5-fluorouracil-related gene modules, and introduced five different machine learning algorithms to complete the task of variable selection, including gradient boosting machine (GBM), least absolute shrinkage and selection operator (LASSO) regression, support vector machines (SVM), Decision Trees, and Random Forest. The intersection of genes selected by each machine learning algorithm was used for stepwise regression analysis in multiple linear regression to generate a fitting regression model to evaluate the risk of samples in different HCC cohorts.

### Nomogram construction

This study integrated age, gender, T stage, stage, grade, and RiskScore information, and performed univariate COX and multivariate COX analyses to determine independent prognostic factors which influenced the prognosis of HCC. Based on these independent prognostic factors, we developed a nomogram for predicting HCC survival. Based on the actual and predicted survival outcomes, we developed calibration curves to validate the predictive power of the nomogram. In addition, we also graphed decision curves to determine the prognostic guidance value of the nomogram and RiskScore.

### Cell culture and transient transfection

HCC cell lines including Hep3B2.1-7 and Huh-7 were obtained from COBIOER (Nanjing, China). Hep3B2.1-7 and Huh-7 cells were cultured in DMEM F12 with 10% FBS (Gibco, Thermo Fisher, USA). Human liver epithelial cells (THLE-3) were obtained from ATCC (Manassas, VA, USA) and stored in BEGM (Lonza, Walkersville). Cells were grown at 37°C in a humidified environment containing 5% CO_2_.

### Quantitative reverse transcription polymerase chain reaction (qRT-PCR)

TRIzol reagent (Thermo Fisher, USA) was used to extract total RNA from the Hep3B2.1-7, Huh-7, and THLE-3 cell lines. Using FastStart Universal SYBR Green Master (Roche, Switzerland), quantitative reverse transcription polymerase chain reaction (qRT-PCR) was performed on the RNA extracted from each sample (2 μg) on a LightCycler 480 PCR System (Roche, USA). The cDNA was utilized as a template with a reaction volume of 20 μl (2 μl of cDNA template, 10 μl of PCR mixture, 0.5 μl of forward and reverse primers, and an appropriate water volume). The following procedures were utilized for the PCR reactions: cycling conditions started with an initial DNA denaturation phase at 95°C for 30 s, followed by 45 cycles at 94°C for 15 s, 56°C for 30 s, and 72°C for 20 s. Three separate analyses were performed on each sample. Based on the 2^−ΔΔCT^ method, data from the threshold cycle (CT) were obtained and standardized to the levels of GAPDH in each sample. The expression levels of mRNA were compared to controls obtained from normal tissues. Sequence lists of primer pairs for the target genes are summarized in Supplementary Table S1.

### Statistical analysis

All statistical analysis and verification were conducted in the R code. A chi-squared test was adopted to detect differences in clinical characteristics between subtypes. The survival of the samples was presented by the Kaplan–Meier curve. Statistical survival difference was analyzed using the log-rank test. The time-dependent receiver operating characteristic (ROC) curve and the area under the curve (AUC) of the risk layering tool were generated and calculated using the timeROC package. *p* < 0.05 meant that the difference was statistically significant. For the results of the statistical analyses, ns indicated no significance, * indicated *p* < 0.05, ** indicated *p* < 0.01, *** indicated *p* < 0.001, and **** indicated *p* < 0.0001.

## Results

### Identification of the gene module most related to 5-fluorouracil

**FIGURE 1:**
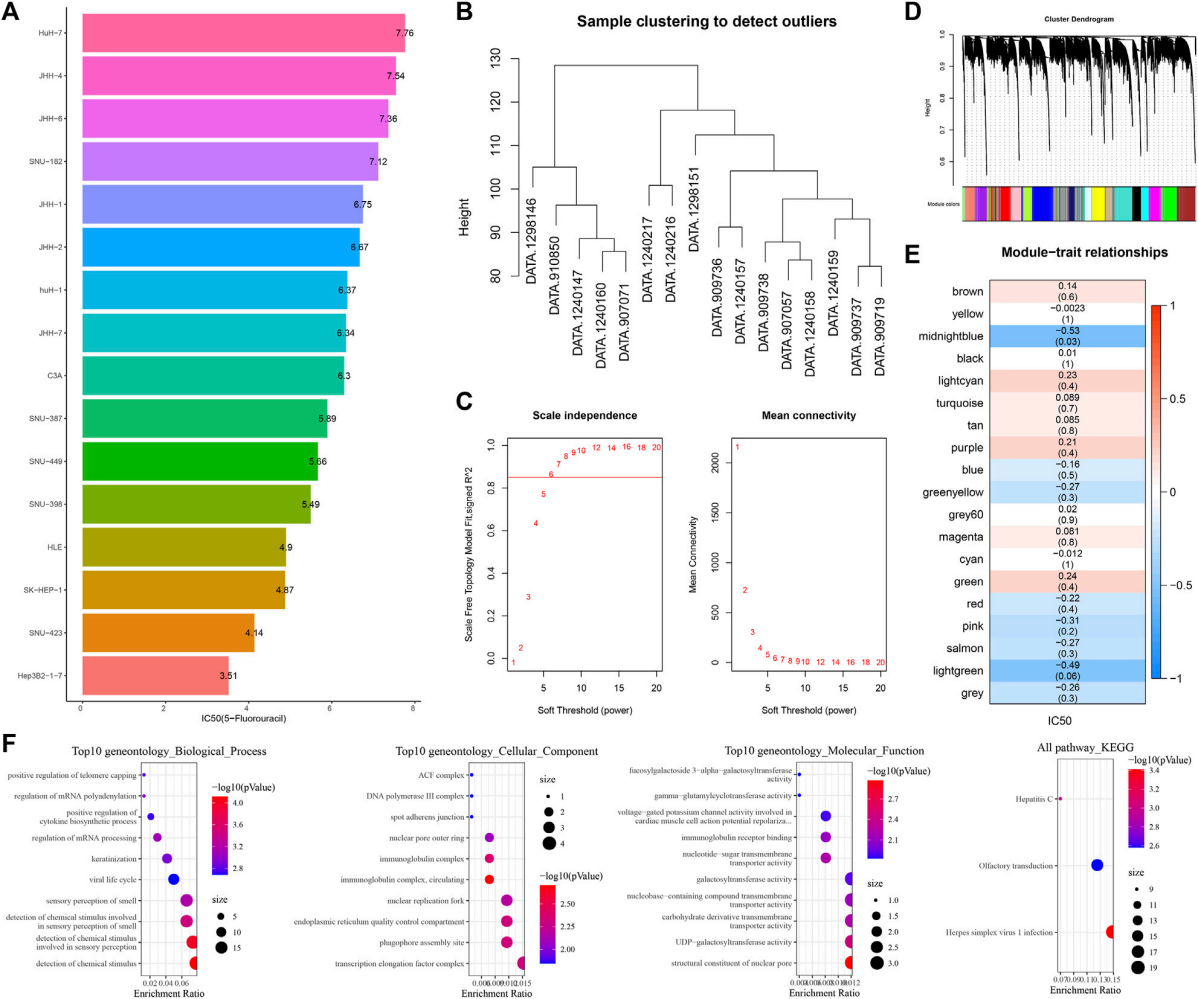
Identification of the gene module most related to 5-fluorouracil. **(A)** Bar chart shows the sensitivity of 5-fluorouracil in different hepatoma cell lines. **(B)** Clustering tree of cell samples from GDSC2. **(C)** Average connectivity corresponding to the scale-free fitting index and each soft threshold. **(D)** Cluster tree of genes in all hepatoma cell lines from GDSC2. **(E)** Correlation analysis of 19 clustered modules with 5-fluorouracil IC_50_. The upper numbers represented correlation coefficients, and the lower numbers represented statistical *p*-values. **(F)** GO entries and KEGG pathways of gene enrichment in the midnight blue module.

We initially examined the sensitivity of different HCC cell lines to 5-fluorouracil. The IC_50_ value of 5-fluorouracil was the lowest in Hep3B2-1-7 cells and the highest in HuH-7 cells, meaning that Hep3B2-1-7 cells were the most sensitive to 5-fluorouracil and HuH-7 cells had the strongest resistance to 5-fluorouracil (Figure 1A). All cell samples from GDSC2 were clustered (Figure 1B). The soft-threshold power satisfying the scale-free topology of the network was 6, the corresponding R2 value was 0.86, and the average connectivity was very close to 0 (Figure 1C). Next, all genes were clustered into 19 interacting modules (Figure 1D). Among the 19 clustering modules, their correlations with 5-fluorouracil resistance were analyzed. The result showed that midnight blue was the module with the highest significant negative correlation with 5-fluorouracil sensitivity (Figure 1E). We analyzed GO and KEGG annotation of genes within the midnight blue module. Biological process was annotated to regulation of mRNA processing, regulation of mRNA polyadenylation, and positive regulation of telomere capping in GO terms. The protein products of these genes might be the components of the transcription elongation factor complex, DNA polymerase III complex (Figure 1F).

### HCC was classified by identifying hub genes in the midnight blue module

**FIGURE 2:**
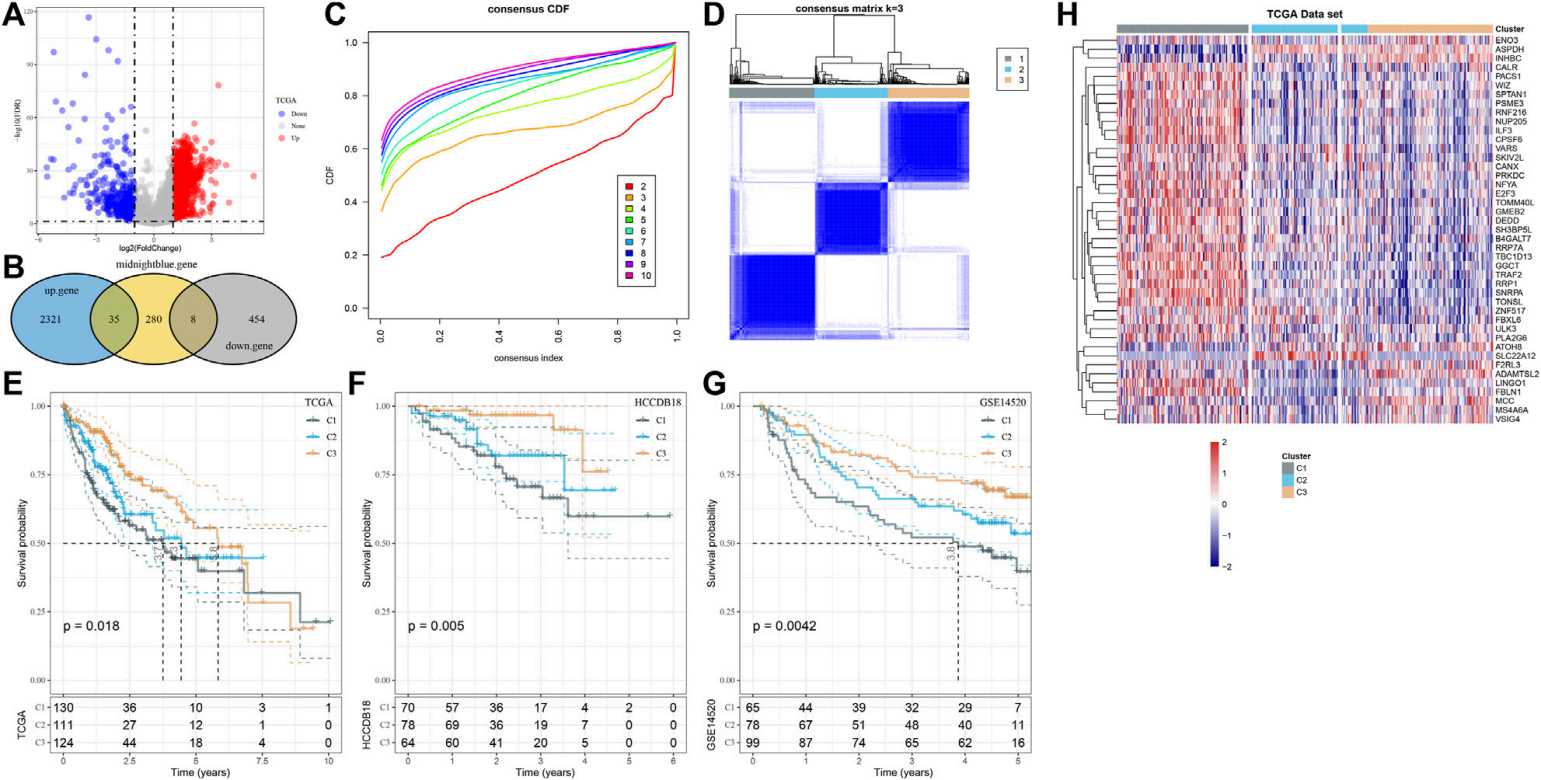
HCC was classified by identifying hub genes in the midnight blue module. **(A)** Difference analysis of log2 (TPM+1) of TCGA-LIHC between normal and HCC tissues. **(B)** The Hub gene of the midnight blue module was identified by overlap analysis of differential genes and the midnight blue module. **(C)** CDF plot displays consensus distributions for each k-value. **(D)** Consensus matrix shows the clustering partition of k = 3. The blue color represented the distance-based similarity between the samples. **(E–G)** Survival curves of three clusters in TCGA-LIHC, HCCB18, and GSE145203 cohorts. **(H)** Expression of 43 genes is shown in the form of a heatmap.

The differences between normal tissues and HCC tissues of log2 (TPM+1) of TCGA-LIHC were analyzed. A total of 2,356 genes with log2 (Fold Change) > 1 and FDR <0.05, as well as 462 genes with log2 (Fold Change) <-1 and FDR <0.05, were identified (Figure 2A), and 35 upregulated and 8 downregulated DEGs were also detected from the midnight blue module (Figure 2B). The samples of TCGA-LIHC were clustered according to the expression of the aforementioned 43 genes. The CDF plot helps in finding the k-value that reached the approximate maximum value of 3 (Figure 2C). The consensus matrix showed the clustering partition was k = 3 (Figure 2D). Complete separation of survival curves and overall survival (OS) of the three clusters in the detected TCGA-LIHC, HCCB18, and GSE145203 cohorts had significant differences among subtypes. Specifically, C3 had the most satisfactory survival outcome when compared with C1 and C2, while C1 had the shortest OS (Figures 2E–G). Next, the expression of 43 genes was also shown in a heatmap, which demonstrated that most genes were highly expressed in C1 than C3 and C2 (Figure 2H).

### Clinical and genomic alteration features of molecular subtypes

**FIGURE 3:**
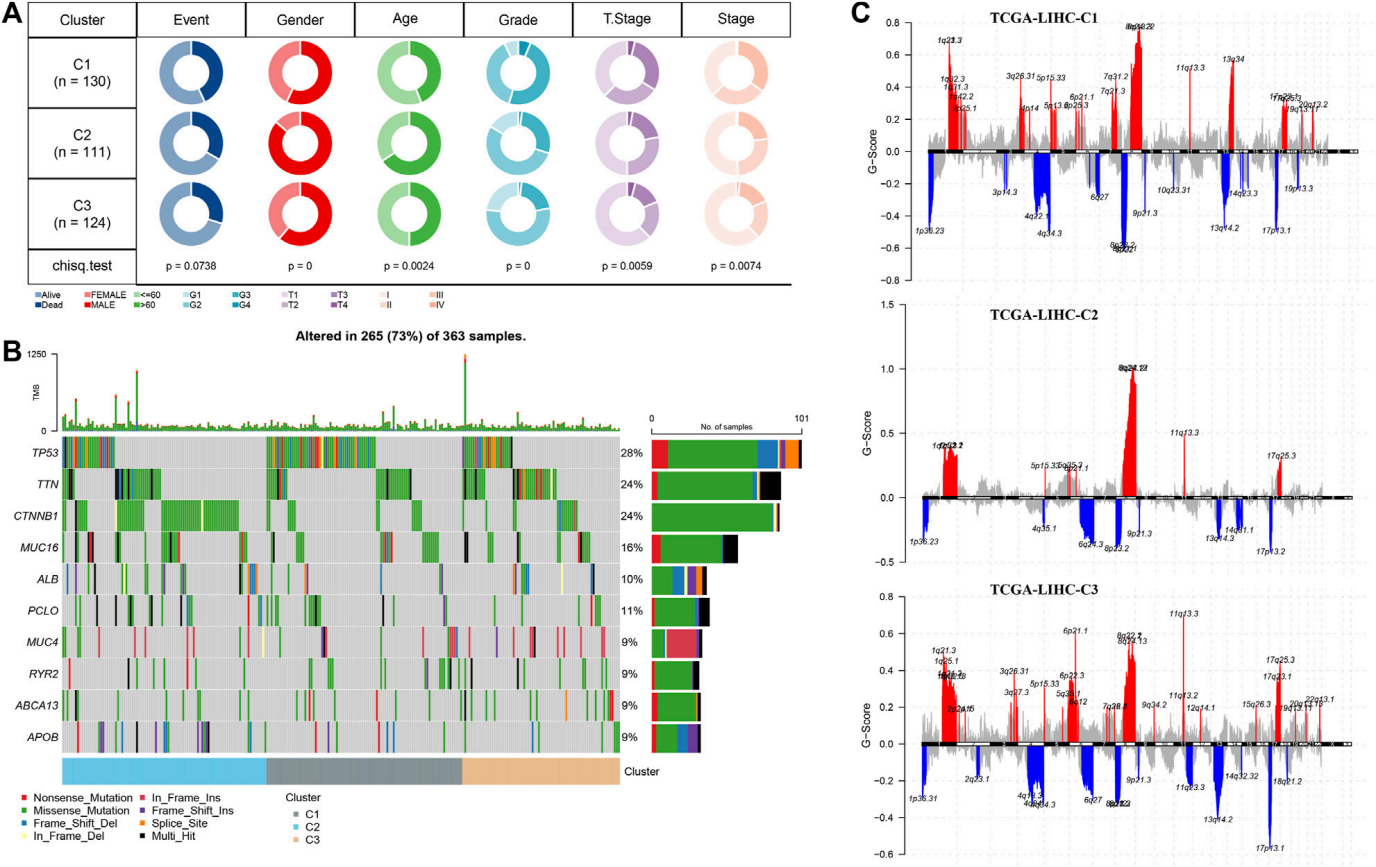
Clinical and genomic alteration features of molecular subtypes **(A)** Waterfall map of somatic mutation in three subtypes. **(B)** Manhattan plot shows the CNV situation of each subtype at the chromosomal level. **(C)** Analysis and comparison of clinical features of three subtypes. The red represents the q-arm of the chromosome, and the blue represents the p-arm of the chromosome.

Here, in the C1, C2, and C3 subtypes, we analyzed the status of genomic variations. The TP53 mutation rate of C1 was also significantly higher than that of C2 and C3. The mutation rate of CTNNB1 in C2 was the highest, which was significantly higher than that of C1 and C3. The mutation rate of TTN in C3 was the highest, which was significantly higher than that of C1 and C2 (Figure 3A). From the Manhattan plot, we observed the CNV of each subtype at the chromosomal level, the number of high-level DNA copies amplified, and deleted in C2 was significantly less than that in C1 and C3 (Figure 3B). Comparison of the clinical characteristics of the three subtypes showed that there were more male subject samples than female subject samples in each subtype. Differences in sex, age distribution and grade, and T stage and stage characteristics were statistically significant among the three subtypes. Compared with the other two clusters, C2 had the highest proportion of male subjects and samples aged over 60 years old. C1 with the shortest OS was characterized by a high proportion of clinical grade and later T stage and stage (Figure 3C).

### Immune filtration mode for three clusters

**FIGURE 4:**
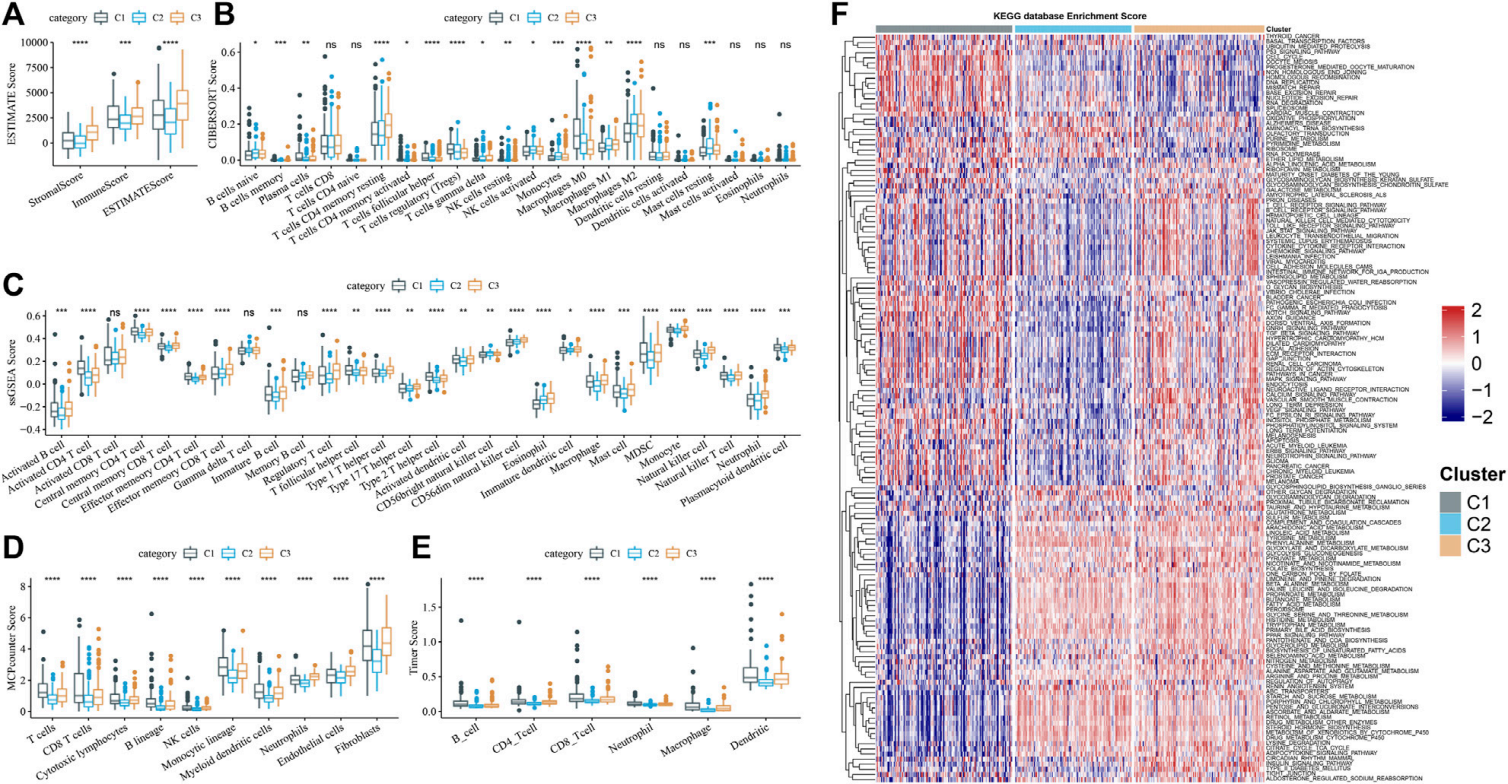
Immune filtration mode for three clusters **(A)** Subtype stromal score, immune score, and ESTIMATE score obtained from running ESTIMATE. **(B–E)** Enrichment differences among subtypes of immune cells judged by CIBERSORT, ssGSEA, MCPcounter, and TIMER. **(F)** Differences in enrichment of pathways in clusters were archived in a heatmap.

By running ESTIMATE, the ESTIMATE score, stromal score, and immune score of each cluster were calculated, which showed significant differences among the three clusters, and the level was the lowest in C2 (Figure 4A). Of the 22 immune cells provided by CIBERSORT, 15 showed significant differences in infiltration among three subtypes (Figure 4B). Memory cells, immunosuppressive cells (regulatory T cells (Treg) and myeloid-derived suppressor cells (MDSC)), and cytotoxic cells (CD8 T cells, natural killer (NK) cells, and NK T cells) identified in 28 TIL subpopulations showed differential infiltration among the three subtypes, and almost all of them had the least infiltration in C2 (Figure 4C). Combining the results of MCPcounter and TIMER analysis, the infiltration of CD4 T cell, T cells, B cells, macrophage, neutrophils, CD8 T cells, endothelial cells, and dendritic cells, and fibroblasts in C2 was significantly lower than that in C1 and C3 (Figures 4D, E). The differences in enrichment of pathways in clusters were archived in a heatmap, from which we could observe that the enrichment level of most pathways relevant to metabolism decreased in C1, such as linoleic acid metabolism, tyrosine metabolism, phenylalanine metabolism, and pyruvate metabolism (Figure 4F).

### Selection and verification of genes suitable for constructing a risk model in the midnight blue module

**FIGURE 5:**
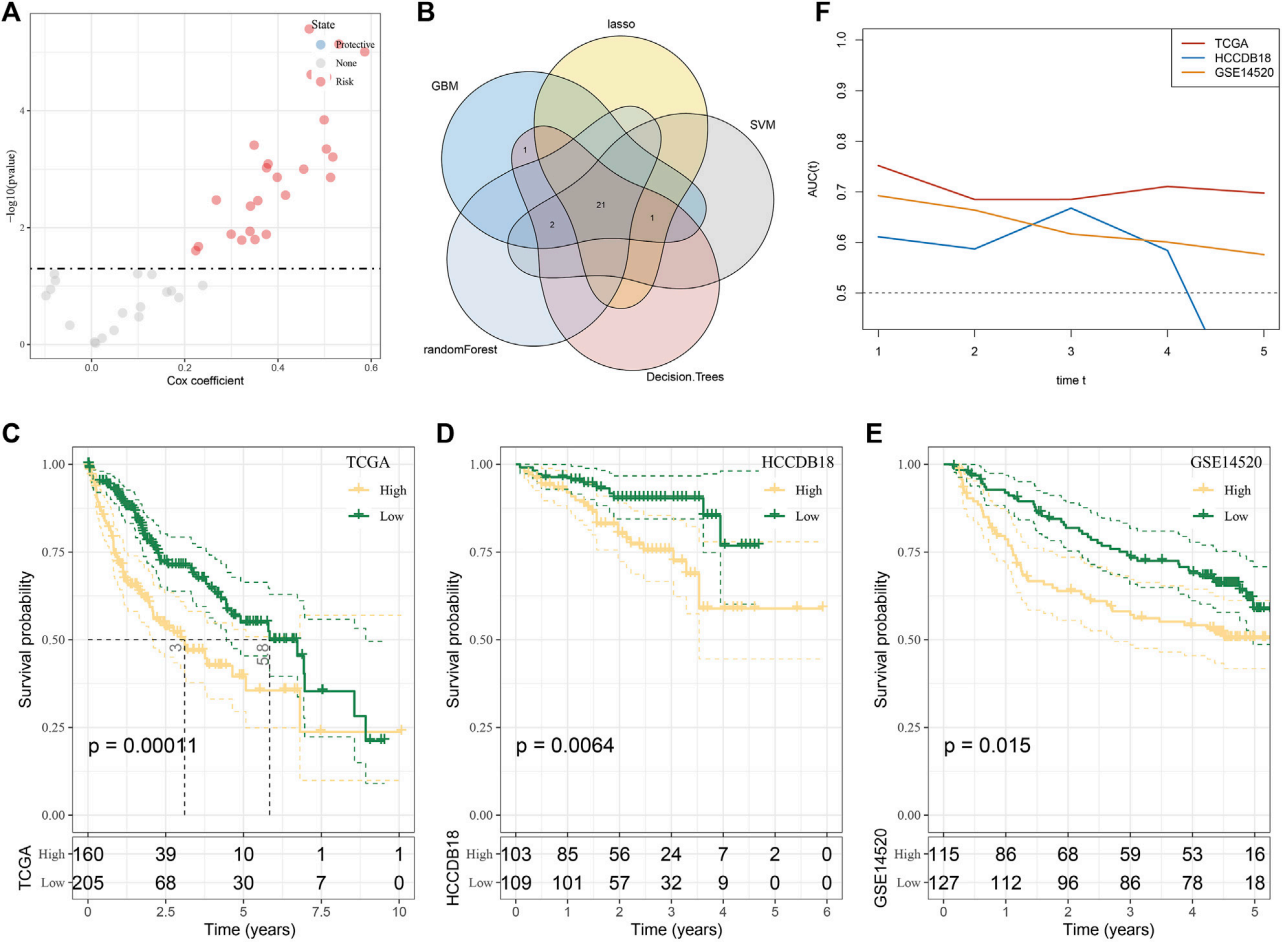
Selection and verification of genes suitable for constructing a risk model in the midnight blue module. **(A)** Volcanic map showing univariate Cox regression analysis for 43 genes. **(B)** Intersection of genes selected by LASSO regression, GBM, SVM, random forest, and decision tree. **(C–E)** Survival stratification curve of the regression model in the test set TCGA-LIHC and two independent verification sets, namely, HCCDB18 and GSE14520. **(F)** ROC curve of regression model in predicting 1–5 years survival of cases in TCGA-LIHC and GSE14520.

In the midnight blue module, a total of 43 genes were identified as hub genes, and 25 HCC prognostic genes were screened from these hub genes by univariate Cox regression analysis (Figure 5A). Machine learning models of these genes were established based on machine learning methods, including LASSO regression, GBM, SVM, Random Forest, and Decision Tree. A total of 21 genes belonged to the intersection of five machine learning models (Figure 5B). The stepwise regression method screened five genes from the 21 genes suitable for constructing a risk model, including *TOMM40L*, *SNRPA*, *ILF3*, *CPSF6* and *NUP205*. The risk coefficient of each gene was obtained from multivariate Cox regression analysis, and a fitted regression model was generated: RiskScore = 0.293*TOMM40L+0.558*SNRPA-0.823*ILF3+0.493*CPSF6+0.464*NUP205. Regression models were used to calculate risk scores in the test set TCGA-LIHC and two independent verification sets HCCDB18 and GSE14520. A significant negative correlation with the sample OS in their cohorts was found, with the patients of a high-risk score showing a shorter survival time (Figures 5C–E). Meanwhile, the regression model showed stability and availability in predicting 1–5 year(s) OS of cases in TCGA-LIHC and GSE14520 (Figure 5F).

### Regression model was an independent predictor of the prognosis and clinical characteristics of HCC

**FIGURE 6:**
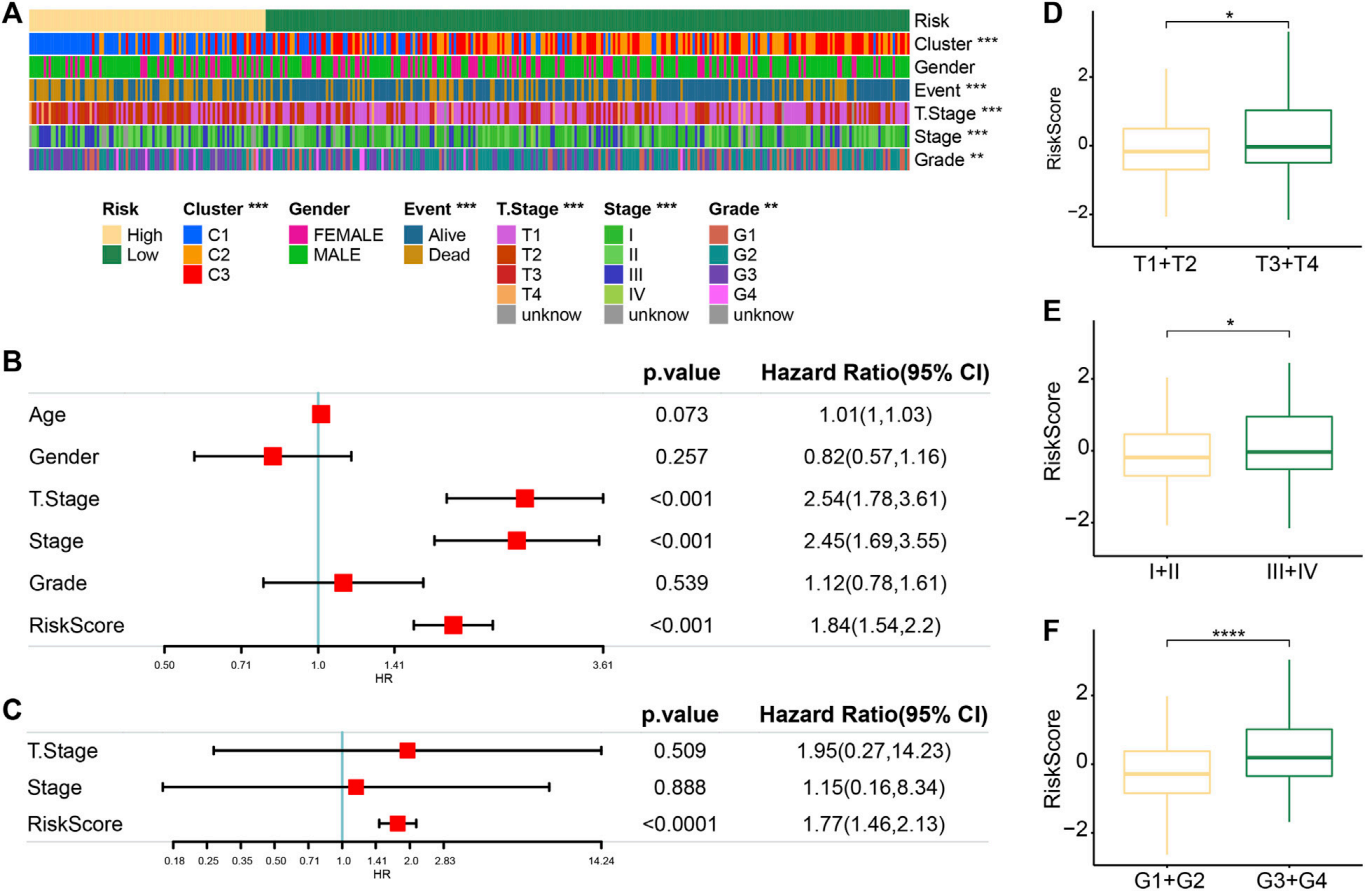
Regression model was an independent predictor of prognosis and clinical characteristics of HCC. **(A)** Heatmaps of clinical traits corresponding to risk score. **(B)** Univariate Cox regression analysis of the risk score and clinical traits. **(C)** Multivariate Cox regression analysis of prognostic traits of HCC based on univariate Cox regression analysis. **(D)** Difference in the risk score between samples stratified according to the T stage. **(E)** Differences in the risk score between samples stratified by stage. **(F)** Correlation between the grade and the risk score of the sample.

The heatmap of clinical characteristics corresponding to risk score was drawn. Differences in molecular subtypes, survival status, T stage and stage, and grade proportion were statistically significant between low-risk and high-risk groups. The high-risk group was characterized by a high C1 ratio, high mortality rate, later T stage and stage, and grade distribution, while these clinical traits in the low-risk group were significantly weaker (Figure 6A). The actual univariate Cox regression analysis showed that the risk score and T stage and stage were significantly correlated with HCC prognosis. The risk score was identified as an independent prognostic index of HCC by Multivariate Cox regression analysis (Figures 6B, C). Synthesizing information on T Stage, Stage, and RiskScore, we constructed the nomogram for assessing clinical outcomes of HCC patients at 1, 3, and 5 years (Supplementary Figure S1A). The calibration curves showed that the predicted clinical outcomes fit well with the actual observed clinical outcomes and that the nomogram had a good predictive value (Supplementary Figure S1B). In addition, the decision curve also showed that there is an excellent applicability of the nomogram and RiskScore in assessing clinical outcomes in HCC (Supplementary Figure S1C). The risk score of the T3–T4 stage, stage Ⅲ–Ⅳ, and G3–G4 samples was significantly higher than that of the T1–T2 stage, stage Ⅰ–Ⅱ, and G1–G2 samples, respectively (Figures 6D–F).

### Relationship between the risk score and immune infiltration

**FIGURE 7:**
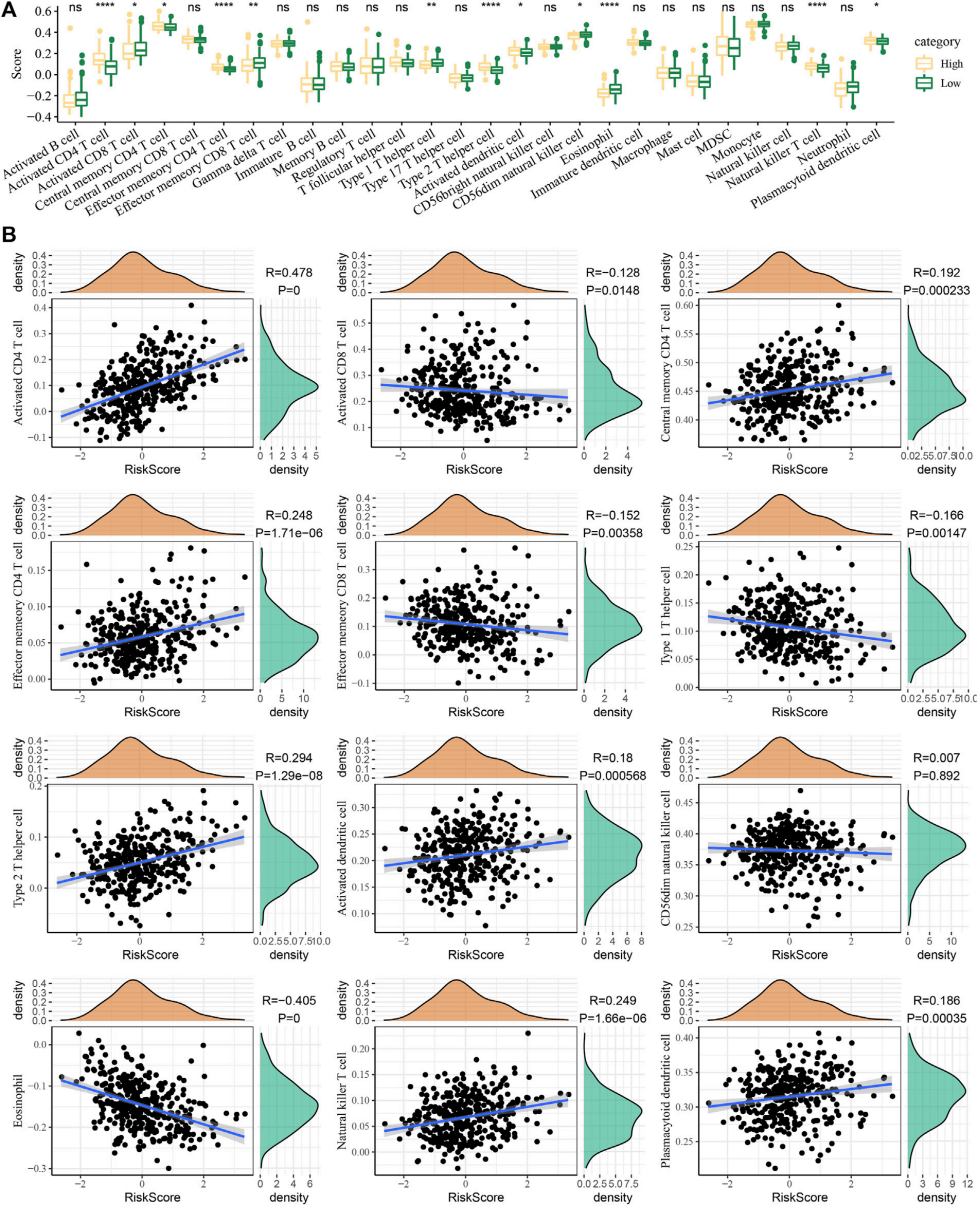
Relationship between the risk score and immune infiltration. **(A)** Immune cell infiltration was evaluated according to the risk score stratification. **(B)** Correlation of the ssGSEA score of the risk score and immune cells.

The degree of immune cell infiltration was evaluated according to the risk score. We detected that the cells with the most different degrees of infiltration in the high-risk and low-risk groups were type 2 T helper cells, central memory CD4 T cells, type 1 T helper cells, plasmacytoid dendritic cells, effector memory CD4 T cells, activated CD4 T cells, activated CD8 T cells, eosinophils, natural killer T cells, CD56 dim natural killer cells, activated dendritic cells, and effector memory CD8 T cells (Figure 7A). The ssGSEA score correlation analysis of the risk score and immune cells showed that the degree of correlation between the 12 cells had infiltration and risk score differences between the high-risk and low-risk groups. The correlation between risk score and CD56 dim natural killer cells was almost negligible, and the other 11 cells showed a significant correlation with risk score. Among them, effector memory CD4 T cells, activated CD4 T cells, natural killer T cells, plasmacytoid dendritic cells, type 2 T helper cells, central memory CD4 T cells, and activated dendritic cells showed a significant positive correlation with the risk score, while activated CD8 T cells, effector memory CD8 T cells and type 1 T helper cells, and eosinophils were significantly negatively correlated with the risk sore (Figure 7B).

### PCR validation of RiskScore

**FIGURE 8:**
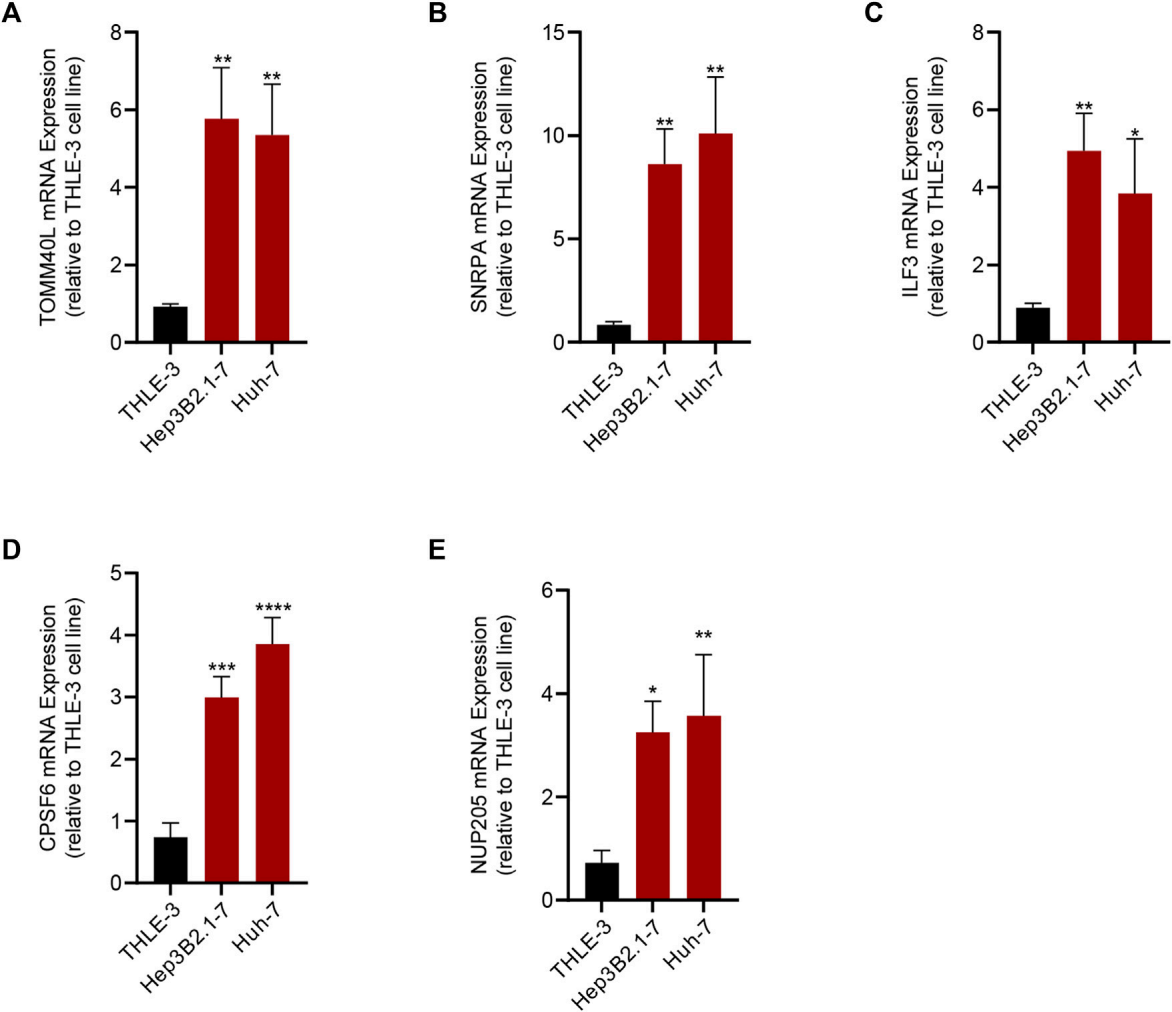
Results of qRT-PCR of the five genes that composed the RiskScore. **(A)** TOMM40L; **(B)** SNRPA; **(C)** ILF3; **(D)** CPSF6; **(E)** NUP205.

To verify the reliability of the RiskScore, we detected the expression of the five genes by PCR. The results of PCR corroborated the reliability of the RiskScore, and we found that *TOMM40L*, *SNRPA*, *ILF3*, *CPSF6*, and *NUP205* were significantly upregulated in the HCC cell lines Hep3B2.1-7 and Huh-7 compared to human normal liver epithelial cells THLE-3 (Figures 8A–E).

## Discussion

The large-scale drug genome cell line database has in-depth multi-group characterization and extensive pharmacological characteristics of human cancer cell lines, and is an important tool to reveal the potential mechanism of inducing drug sensitivity of anticancer drug compounds (Kusch and Schuppert, 2020). This study explored how HCC heterogeneity of HCC was affected by the molecules related to 5-FU sensitivity based on the expression profile of the cancer cell line in the largest public resource and the sensitivity data on 5-FU, a commonly used cancer chemotherapy drug. The WGCNA analysis identified the midnight blue gene module with the highest correlation with 5-FU sensitivity and 43 hub genes in the module. Three subgroups of HCC were defined according to their expression. This classification supported most of the genomic variation, TME, and pathological pathway heterogeneity observed in HCC.

Currently, 5-FU was the mainstream tumor chemotherapeutic agent (Blondy et al., 2020; Vodenkova et al., 2020). Accumulating evidence illustrated that 5-FU exhibited cytotoxicity by binding to DNA or RNA and modulating DNA synthesis-induced cell cycle disruption or apoptosis (Sethy and Kundu, 2021). Cell cycle abnormalities are typical in tumor cells, and inhibition of the tumor cell cycle is essential for suppressing cell proliferation and spreading, and even restoring immune cell surveillance functions (Liu et al., 2022). Based on the results of GO and KEGG annotations, we showed that midnight blue endogenous genes were closely associated not only with mRNA processing, regulation of mRNA polyadenylation, and positive regulation of the telomere capping pathway but also with the transcription elongation factor complex, DNA polymerase III complex synthesis. 5-FU disrupted the homologous recombination repair process in cells, leading to DNA damage and inhibition of proliferation in tumor cells (Srinivas et al., 2015). The mRNA processing, regulation of mRNA polyadenylation, and positive regulation of telomere capping were important regulators in the cell cycle in normal cells. The genes within the midnight blue module were recognized as gene modules sensitive to 5-FU treatment, suggesting the possibility that HCC might act through these biological processes when treated with 5-FU.

In terms of survival outcomes, C3 possessed the most satisfied survival outcome when compared with C1 and C2, and C1 demonstrated the shortest OS. Each subgroup also showed its own unique clinical characteristics, C2 had the highest proportion of male subjects and samples aged over 60 years old than C1 and C3. C1 with the shortest OS was characterized by a high proportion of clinical grade and later T stage and stage, and most metabolic pathways of this subtype were significantly inhibited. These bad characteristics were also reflected in OS, and C1 had the most unfavorable survival outcome. The gene with the highest mutation rate was TP53 in C1, CTNNB1 in C2, and TTN in C3. This indicated that C1 was a tumorigenesis subtype driven by TP53 mutation, C2 was a tumorigenesis subtype driven by CTNNB1 mutation, and C3 was a tumorigenesis subtype driven by TTN mutation. TP53 mutations and CTNNB1 mutations were most common in HCC. In a study by Gao et al. (2019), HCC patients characterized by TP53 mutations had a dysregulated cell cycle and DNA damage repair pathways, and TP53 was the gene with the highest mutation frequency. Low TP53 levels inhibited HCC development. Significant activation of metabolic reprogramming was demonstrated in patients enriched with CTNNB1 mutations. This phenomenon promoted glycolytic metabolic intensity and cell proliferation in HCC. It was also confirmed that the frequency of the R249S mutation in TP53 revealed the risk of HCC, and TP53 deletion increased the viability of hepatocellular carcinoma cells and the trend of poor prognosis (Lam et al., 2022). Interestingly, the C3 isoform might be a TTN mutation-driven molecular subtype that exhibited a high mutational profile. However, Kunadirek et al. (2021) noted that TTN mutations in blood predicted unfavorable prognostic status in HCC patients. The results of survival analysis in this study demonstrated that the C1 subtype predicted had an unfavorable prognosis, C2 subtype had a moderate prognosis, and C3 subtype had the best prognosis. However, we must point out that there were differences in sampling between them as tissue samples in our study were different from the blood samples in Kunadirek’s. Second, HCC was a highly heterogeneous tumor both in terms of genomic composition and gene mutations (Jeng et al., 2015). The research challenges posed by the heterogeneity remain difficult to resolve. Overall, the C1 subtype in our study was enriched with TP53 mutations, and patients enriched with TP53 mutations tended to have unfavorable survival outcomes. Patients enriched with CTNNB1 mutations showed significant metabolic reprogramming activity, and inhibition of glycolytic signaling could be considered to target the C2 subtype to improve prognosis. Different treatment options could be considered for patients with C1 and C2 subtypes to achieve precision cancer treatment.

Personalized treatment for HCC patients has been increasingly recognized and applied in the clinical field. The development of risk models represents an important step toward personalized HCC monitoring. Although many risk models have been published, few are used in routine nursing to provide information for HCC monitoring decisions (Innes and Nahon, 2023). In this study, five of the 43 hub genes in the midnight blue module were used to develop a risk regression model, which was independent and had strong discriminating power for predicting HCC prognosis and indicating clinical traits. The pathological role and regulatory mechanism of some of them in cancerization have been recognized. SNRPA is a shear factor associated with microvascular invasion and promotes the metastasis of HCC by activating the NOTCH1/Snail pathway mediated by the circSEC62/miR-625–5p axis (Mo et al., 2023). ILF3 is overexpressed in patients with primary colorectal cancer and promotes tumor growth by directly regulating the mRNA stability of *SGOC* gene (Li et al., 2020). *CPSF6* is upregulated in HCC and induces metabolic changes in hepatocytes through the alternative polyadenylation of *NQO1* (Tan et al., 2021). Although the potential effects of these genes on cancer have been reported, the risk model integrating these genes was an innovative exploration and could be used as an indicator of the prognosis of HCC.

In summary, this study classified HCC subtypes based on the sensitivity to 5-FU. The results supported the prognostic differences observed in HCC and the heterogeneity of most genomic variations, TME, and pathological pathways. This study also provided an independent prognostic risk regression model integrated with five 5-FU-related genes, contributing to the study of individualized HCC monitoring.

## Data Availability

The original contributions presented in the study are included in the article/Supplementary Material; further inquiries can be directed to the corresponding authors.

## Abbreviations

5-FU, 5-fluorouracil; HCC, hepatocellular carcinoma; WGCNA, weighted gene co-expression network analysis; OS, overall survival; TS, thymidylate synthase; TME, tumor microenvironment; TCGA, The Cancer Genome Atlas; GEO, Gene Expression Omnibus; GDSC, Genomics of Drug Sensitivity in Cancer; DEG, differentially expressed gene; CDF, cumulative distribution function; SNV, single-nucleotide variant; CNV, copy-number variant; GBM, gradient boosting machine; LASSO, least absolute shrinkage and selection operator; SVM, support vector machines; ROC, receiver operating characteristic; AUC, area under the curve; Treg, regulatory T cell; MDSC, myeloid-derived suppressor cell.
