# Supplementary material for: Heterogeneity characterization of hepatocellular carcinoma based on the sensitivity to 5-fluorouracil and development of a prognostic regression model
Source: Front Pharmacol. 2023 Sep 7;14:1252805. doi: 10.3389/fphar.2023.1252805 (PMC10512943; doi:10.3389/fphar.2023.1252805)
Supplement: Supplementary file 1 [file Table1.DOCX]

Supplementary Figure 1 A nomogram composed of independent prognostic factors for HCC.

(A) Nomogram composed of T Stage, Stage, and RiskScore. (B) Calibration curve. (C) Decision curve.
