# Supplementary material for: Heterogeneity characterization of hepatocellular carcinoma based on the sensitivity to 5-fluorouracil and development of a prognostic regression model
Source: Front Pharmacol. 2023 Sep 7;14:1252805. doi: 10.3389/fphar.2023.1252805 (PMC10512943; doi:10.3389/fphar.2023.1252805)
Supplement: Supplementary file 2 [file Table2.DOCX]

Table 1: Primer information for six prognostic genes in RiskScore.

| Gene | Forward primer sequence (5’-3’) | Reverse primer sequence (5’-3’) |
| --- | --- | --- |
| TOMM40L | AGGTCTTGCTCCTCTTGGCAGA | TAGGGTCAGAGTGGCTGTGTAG |
| SNRPA | ATCCAGGTGCTGGTTTGCCAAC | GGATCTGACCAGACTGCAGCAA |
| ILF3 | CCTGACAAAGCACGGCAAGAAC | CCAGCACCTTGGAACTTCTGTC |
| CPSF6 | GCCTACATCAGATAGCCGAGGT | GCTTCACTCAATGGCGTTCTTGC |
| NUP205 | GATCCAGGAGTGTTAGGTTGCC | GACCTGGTAACATAGCTCAGCC |
| GAPDH | GTCTCCTCTGACTTCAACAGCG | ACCACCCTGTTGCTGTAGCCAA |
