# Supplementary material for: Heterogeneity characterization of hepatocellular carcinoma based on the sensitivity to 5-fluorouracil and development of a prognostic regression model
Source: Front Pharmacol. 2023 Sep 7;14:1252805. doi: 10.3389/fphar.2023.1252805 (PMC10512943; doi:10.3389/fphar.2023.1252805)
Supplement: Supplementary file 3 [file Table3.DOCX]

Table 1 Information on overlapping DEGs

| Symbol | Type |
| --- | --- |
| TOMM40L | upregulated |
| B4GALT7 | upregulated |
| ZNF517 | upregulated |
| DEDD | upregulated |
| TRAF2 | upregulated |
| SH3BP5L | upregulated |
| SNRPA | upregulated |
| ILF3 | upregulated |
| GMEB2 | upregulated |
| SPTAN1 | upregulated |
| TONSL | upregulated |
| TBC1D13 | upregulated |
| VARS | upregulated |
| RRP1 | upregulated |
| FBXL6 | upregulated |
| SKIV2L | upregulated |
| GGCT | upregulated |
| ULK3 | upregulated |
| CALR | upregulated |
| PLA2G6 | upregulated |
| PSME3 | upregulated |
| WIZ | upregulated |
| RNF216 | upregulated |
| CPSF6 | upregulated |
| CANX | upregulated |
| RRP7A | upregulated |
| PRKDC | upregulated |
| NFYA | upregulated |
| NUP205 | upregulated |
| PACS1 | upregulated |
| F2RL3 | upregulated |
| E2F3 | upregulated |
| LINGO1 | upregulated |
| FBLN1 | upregulated |
| SLC22A12 | upregulated |
| MS4A6A | downregulated |
| ENO3 | downregulated |
| MCC | downregulated |
| ATOH8 | downregulated |
| VSIG4 | downregulated |
| ASPDH | downregulated |
| ADAMTSL2 | downregulated |
| Symbol | Type |
| INHBC | downregulated |
