# Supplementary figures and images for: Heterogeneity characterization of hepatocellular carcinoma based on the sensitivity to 5-fluorouracil and development of a prognostic regression model
Source: Front Pharmacol. 2023 Sep 7;14:1252805. doi: 10.3389/fphar.2023.1252805 (PMC10512943; doi:10.3389/fphar.2023.1252805)

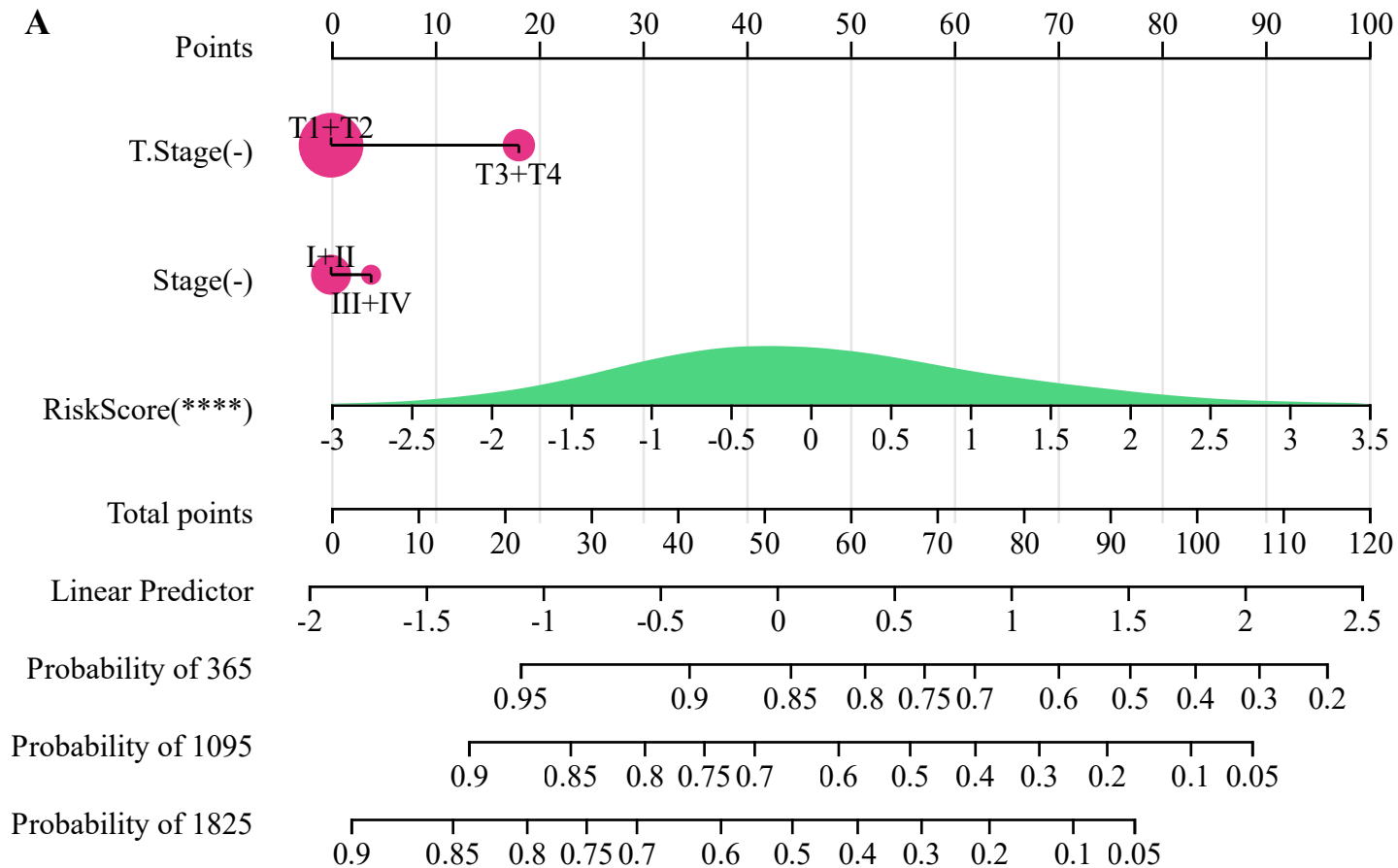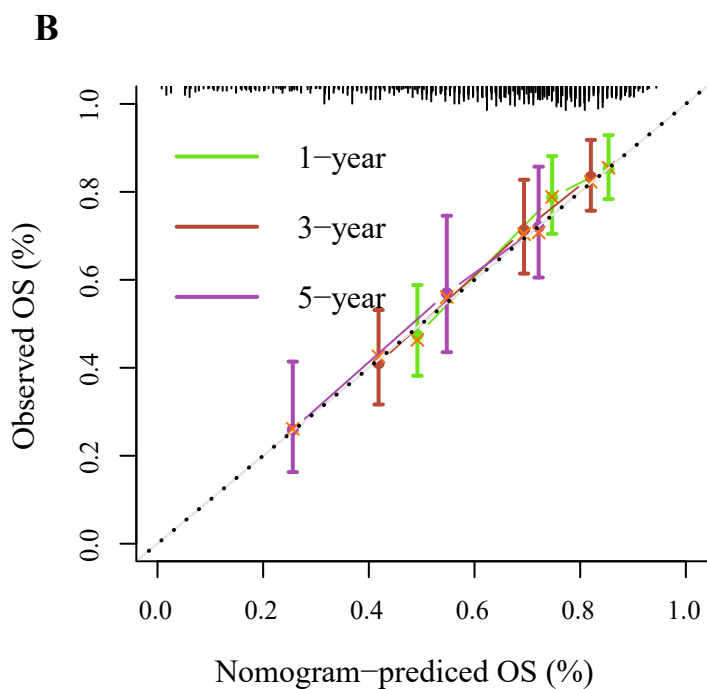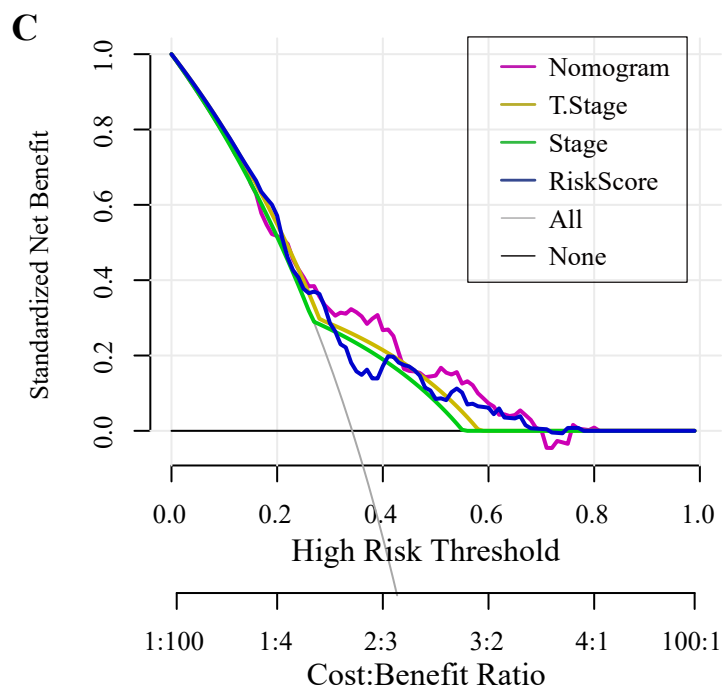

Supplement: Supplementary file 4 [file Image1.PDF]
